# Are Changes in the Mean or Variability of Climate Signals More Important for Long-Term Stochastic Growth Rate?

**DOI:** 10.1371/journal.pone.0063974

**Published:** 2013-05-14

**Authors:** Bernardo García-Carreras, Daniel C. Reuman

**Affiliations:** 1 Imperial College London, Ascot, Berkshire, United Kingdom; 2 Laboratory of Populations, Rockefeller University, New York, New York, United States of America; University of Exeter, United Kingdom

## Abstract

Population dynamics are affected by changes in both the mean and standard deviation of climate, e.g., changes in average temperature are likely to affect populations, but so are changes in the strength of year-to-year temperature variability. The impacts of increases in average temperature are extensively researched, while the impacts of changes in climate variability are less studied. Is the greater attention given to changes in mean environment justified? To help answer this question we developed a simple population model, explicitly linked to an environmental process. We used the model to compare the sensitivities of a population's long-term stochastic growth rate, a measure of fitness, to changes in the mean and standard deviation of the environment. Results are interpreted in light of a comparative analysis of the relative magnitudes of change in means and standard deviations of biologically relevant climate variables in the United States. Results show that changes in the variability of the environment can be more important for many populations. Changes in mean conditions are likely to have a greater impact than changes in variability on populations far from their ideal environment, for example, populations near species range boundaries and potentially of conservation concern. Populations near range centres and close to their ideal environment are more likely to be affected by changes in variability. Among pest and insect disease vectors, as well as species of commercial value, populations likely to be of greatest economic and public health significance are those near species range centers, living in a near-ideal environment for the species. Observed changes in the variability of climate variables may benefit these populations.

## Introduction

Ongoing climate change is most readily characterized by changes in the mean state of climate variables (e.g., increases in mean temperature [1]), and the impacts on ecosystems of changes in mean environmental state are studied closely [2], [3]. However, rising levels of greenhouse gases may also affect climate variability [4]. An increase in variability could also affect populations' fitness [5]–[9]. How do changes in the variability of climate compare to changes in the mean values of climate variables in terms of the importance of their impacts on populations? To help answer this question, we here consider the simplest possible population model that can be linked to an environmental process.

Changes in mean climate have been well documented (e.g., [1]), and while changes in variability have received less attention, they have been studied at different temporal resolutions (e.g., daily [10]; monthly [11], [12]; seasonal [13]; annual [4], [14]), using both empirical data michaelsetal [15]–[17], and forecasts from a range of models [18]–[20]. These studies show that for some temporal resolutions, the variability of climate is changing.

Environmental variables affect annual population growth rates and vital rates such as survival probabilities and fecundity rates; it is through these rates that changes in the mean or variability of climate can affect long-term population growth rates. Determining the consequences of climatic changes on population growth therefore requires understanding the relationship between environment and annual growth and vital rates, i.e., how an environmental signal is translated into biological processes [3], [21]. For ectotherms, which comprise over 99% of all species [22], temperature alters the speed at which individuals pass through life stages, thereby influencing population growth rate [23]–[25]. In ectotherms, the relationship between temperature and annual net population growth rate (henceforth called the response function) typically has a single peak; there is an ideal temperature that maximizes the population's performance [21], [23], [26]–[32]. An argument for a single-peaked response function can also be made for endotherms [33] and other environmental variables such as precipitation [26]. Alternative shapes of functional responses may occasionally be reported in studies, but these can often be considered special cases of the single-peaked response function; we come back to this point in the Discussion. The specific shape of the response function for a species may determine how variability in temperature or another environmental variable affects the long-term population growth rate [34]–[37]. If a response function is log-convex (the log of the function opens up) for the range of an environmental variable that pertains in a locale, then an increase in variability may in fact benefit the population; if the function is log-concave (its log opens down) for the pertinent range of the variable, then variability is detrimental for the population [34], [35].

The response function therefore plays an important role in determining the impacts of climate change on populations. There are several important studies that compare the effects of changes in mean and variability of vital rates on long-term population growth rate (e.g., [3], [38]–[40]). However, changes in the mean environment can modify both the mean and standard deviation of vital rates and annual growth rates, as can changes in the standard deviation of the environment; understanding the relative importance of changes in means and variabilities of vital rates and annual growth rates does not necessarily translate directly to the relative importance of changes in the means and variabilities of environmental variables for long-term growth. *A priori*, the translation from environments to annual growth and vital rates may affect the relative importance of means and standard deviations. This possibility can be investigated by explicitly using response functions to characterize the relative sensitivities of a population to changes in the means and standard deviations of environmental variables.

In addition to examining relative sensitivities, to understand the relative importance for populations of changes in the means and standard deviations of environmental variables, it is also necessary to understand the relative magnitudes of these changes. Even if, hypothetically, a population were more sensitive to changes in the standard deviation of an environmental variable than to changes in the mean of the same variable, if the mean of the variable is changing much more rapidly than the standard deviation, changes in mean may impact the population more. Sensitivities of a population to changes in means and standard deviations of environmental variables must be multiplied by the changes taking place to assess relative importance of the two types of change.

We know of only two studies that incorporate response functions and compare the effects of changes in mean and variability of the environment, as opposed to vital rates, on a population. Van de Pol *et al.* [37] and Jonzén *et al.* [41] parameterized stage-structured stochastic population models using populations of oystercatchers in the Netherlands and red kangaroos in South Australia, respectively. Van de Pol *et al.* concluded that time to extinction is more sensitive to changes in the environment's mean than its standard deviation, a result further magnified by the fact that climate models predict greater changes in mean temperature than in its standard deviation in the Netherlands. Jonzén *et al.* also found sensitivity of population growth to be greater to changes in mean rainfall than to changes in the standard deviation of rainfall, although the two sensitivities were similar enough that changes in standard deviation would still be important unless changes in mean rainfall were much greater than changes in the standard deviation of rainfall.

In this study we aim to compare the effects of changes in mean and variability of inter-annual physical environmental conditions on long-term population growth rate, which we use as a measure of fitness, adopting a simple, strategic approach rather than parameterising a complex model of a single population as in [37], [41]. Both approaches are valuable. We provide a theoretical approach based on an unstructured, annually censused population, which we assume is explicitly linked to an annual environmental variable via a response function. The model is the simplest possible stochastic matrix model, a class of model very widely used for analysis of the growth rates and extinction risks of real populations (e.g., references [42], [43]). We first derive the population long-term stochastic growth rate as a function of the environment and the response function. We then derive the sensitivity of long-term growth rate to changes in environmental mean and variability. Finally, we compare sensitivities to observed changes in the means and standard deviations of several environmental variables likely to influence populations. We provide answers based on our model to the following three questions: (1) Given an increase in the mean or standard deviation of the environment, does the long-term growth rate increase or decrease? (2) If mean and standard deviation are perturbed by the same small amount, which causes the greater impact on the long-term growth rate? (3) What are the relative magnitudes of observed changes in mean and standard deviation of climate variables, and how do these relate to the sensitivities computed in (2) to yield an overall idea of whether changes in climate means or standard deviations are more important for population dynamics? We discuss results in view of currently ongoing climate change, and identify potential consequences for populations of conservation concern as well as pests, disease vectors, and exploited populations. We indicate conceptually why results are likely to generalize from the simple model we employ to more complex models and real populations.

## Methods

### Theory

For 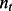 representing the population in year 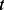, the base model [44] is
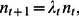
(1)where 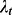 is the net growth rate of the population in year 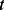. We assume 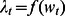, where 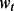 is the physical environmental variable and 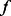 is the response function. Let 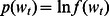 be the log of the response function. For the stochastic model, population size asymptotically approaches a lognormal distribution, with mean 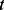 times a quantity denoted 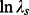 (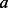 in [45]; ''infinitesimal mean''  in [46]; 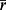 in [47]); 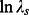 is the long-term stochastic growth rate [42], [45], [48],
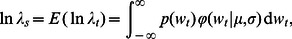
(2)where 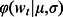 is the probability density function (pdf) of 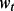, with mean parameter 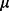 and standard deviation parameter 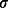 [47], [48]. The integral in equation (2) is the definition of the expected value. Second-order approximations to 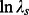 [45], [48] are used, but equation (2) is an exact formula that applies in the case of an unstructured population. The long-term stochastic growth rate 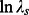 represents the rate at which almost every realization of the population grows [7], [42], [49] and is widely studied as a fitness parameter boyceetal06 and in practical application [42], [43]. The sensitivities of  to changes in mean and standard deviation of the environment are obtained simply by taking the partial derivatives of equation (2) with respect to 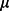 and 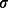, moving the partial derivatives under the integral symbol and applying them to 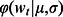. This approach applies generally, for any 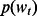.

**Figure 1:**
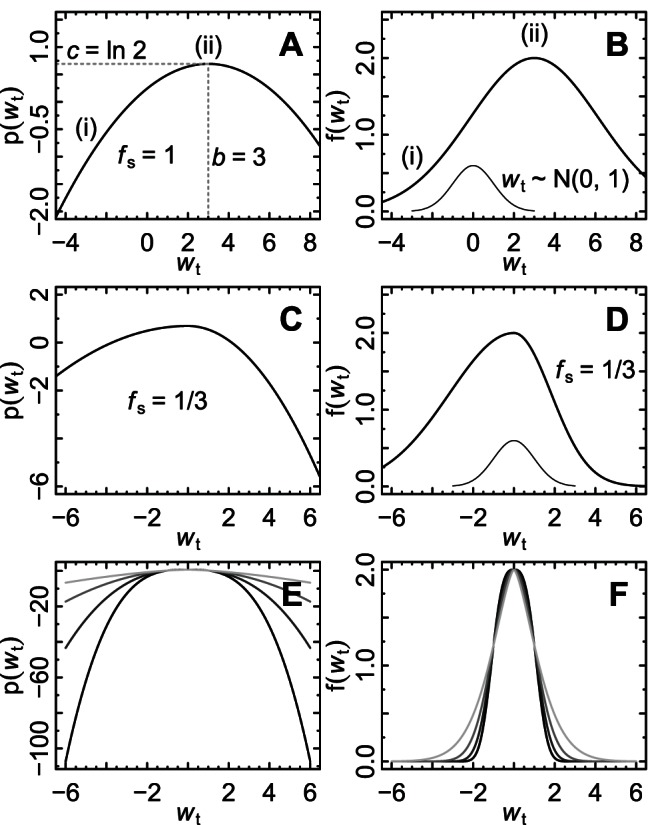
Example log and linear response functions. In the model considered, the response function is the relationship 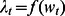 between the net growth rate, 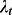, at time 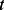, and the environment, 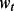, at time 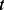. In this figure, we indicate the flexibility of our parameterization of 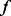 (see main text for details). A log response function 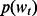 (A) and corresponding linear-scale response function 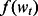 (B) for 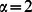, 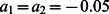, 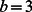, and 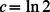. Region (i) represents a suboptimal environment and region (ii) represents an optimal environment. An example is also shown for an asymmetric response function with 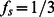 (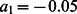, 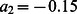) on the log (C) and linear (D) scales, for 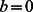, 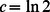, and 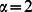. Standard normal distributions (B, D) represent the population's local environment 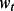. In B, the population is in a suboptimal environment, for instance at the periphery of the species' range. In D the population is close to its ideal environment. Response functions on the linear (E) and log (F) scales for different values of 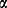, ranging from 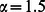 for the light grey, to 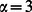 for the black line, for 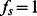, and 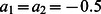. The intermediate values of 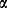 are 2 and 2.5.

For concreteness, we adopt a flexible parameterization for 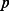. We transform 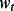 such that its distribution in the focal location is 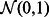 (see Section S1 in File S1). This step should result in no loss of generality for many environmental variables, such as mean annual temperature and rainfall. For some 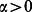, 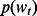 is taken to be 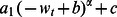 for 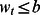 and 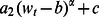 for 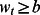 (Figure 1). This function is single peaked. The maximum height of the response function is controlled by 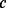. The ideal environment, at which the response function is maximized, is controlled by 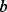; the term ''ideal environment'' is henceforth used to refer simply to the value of 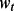 at which 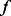 is maximized. The rate of falloff of 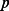 as 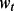 decreases (respectively, increases) from the ideal environment is controlled by 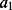 (respectively, 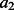); both are taken to be negative. The ratio 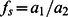 is a measure of asymmetry of the response function around 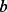; so 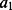 and 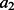 control the rate of falloff of the response function from the ideal environment and their relative magnitude controls symmetry. The functional form or general shape of the falloff is determined by 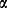 (Figure 1E, F); 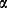 is included as a variable (as opposed to a fixed value such as 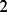) for flexibility, so that response functions of a variety of shapes can be considered. The 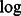 concavity of each half of the response is controlled by 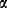, with 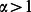 corresponding to 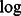-concave response functions and 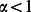 to 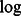-convex ones; 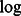-concavity has been important in prior work [34]–[37]. The response function shapes that can be generated with our parameterization (examples in Figure 1) are similar to many reported response functions [23], [26], [28], [29]. The parameterization of 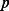 was chosen because it is very flexible, encompassing a wide range of possible relationships between the environment and vital rates, including asymmetries and different rates, functional forms, and 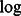-curvatures for falloff of the vital rate from the optimum. The parameter 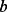 is measured in units equal to the standard deviation of the local environment because we re-scaled 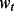 to make it standard normally distributed. Here the term ''local environment'' refers to the distribution of 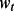. Larger values of 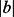 describe populations living in a suboptimal environment (for example, those living in environmental range margins or struggling to adapt to climate change), whereas 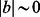 represents populations living in a close-to-ideal environment.

Substituting the above parameterization of 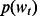 into equation (2), we get 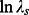 as a function of the parameters that define the log response function,
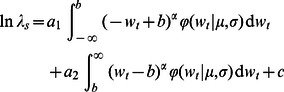
(3)(Section S2 in File S1), where 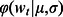 now represents the pdf of the normal distribution with mean 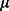 and standard deviation 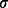. It is straightforward to compute the partial derivatives of 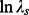 with respect to 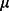 and 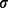 at 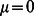 and 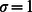 (Sections S3 and S4 in File S1). These are the instantaneous rates of change of 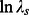 per unit change in 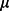 and 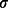 respectively, where the unit of change in 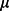 and 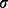 is one standard deviation of 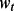. The signs of these sensitivities indicate whether a small increase in mean or standard deviation of the environment increases or decreases 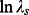. Following the rationale of [37], the relative magnitudes of these sensitivities provide an estimate of whether small changes in environmental mean or standard deviation have a bigger influence on 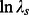.

#### Analysis of climate data

To analyze changes in environmental variables, we downloaded Version 2 of the United States Historical Climatology Network database (USHCN [50], [51]) and extracted annual time series of mean summer and winter temperatures, minimum winter temperatures, maximum summer temperatures, and total spring precipitation for locations in the conterminous United States (Section S5 in File S1). Annual time series were used because our model is more consistent with annually measured populations and environmental variables. We chose weather variables that are likely to be biologically meaningful to populations living in temperate latitudes. The USHCN data were filtered to include only time series that covered the entire 1911–2010 period. Each time series was then split into two periods (1911–1945 and 1976–2010), each of 35 years length. We calculated the mean and standard deviation of the climate variables listed above, for the two periods separately. Prior to calculating the standard deviation, each time period was detrended to remove quadratic and linear trends that could otherwise inflate the amount of variability measured. Because detrending can also remove low-frequency variability, we repeated analyses with linear detrending, and again with no detrending. To approximate normality, square-root precipitation data were used.

Although prior climatological analyses have examined changes in the means and standard deviations of climate variables (e.g., [1], [4], [10]–[17]) these studies have not sought explicitly to compare the relative magnitudes of changes in means and standard deviations for multiple biologically important variables, using the same data for both statistics to ensure comparability. A direct comparison is key for our research purposes. Hansen *et al.* [17] computed means and standard deviations using the same data set, but examined only season-average temperature variables, and used data which represent spatial averages computed over 250 by 250 km or 1200 by 1200 km grid squares. Such low spatial resolution is probably less relevant to many populations than the higher resolution used here.

## Results

### Theoretically Predicted Sensitivities

We now provide answers to questions (1) and (2) posed in the Introduction by considering a simple special case and then by showing the general case produces substantially the same results. The special case is 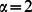 and 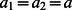 (so 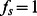). For this special case, the log response function is symmetric (Figure 1A and B) and 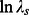 and sensitivities can be calculated entirely analytically:
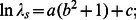
(4)

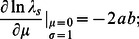
(5)

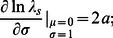
(6)(Section S6 in File S1). The signs of the sensitivities of 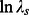 to changes in 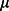 and 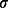 provide an answer to the first question posed in the Introduction: given a change in the mean or standard deviation of the environment, does the growth rate increase or decrease? The sign of the sensitivity of 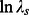 to changes in 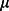 is the same as the sign of 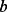, since 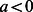; hence any change in the mean environment toward a population's optimum will increase 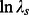, as expected. The sensitivity to changes in 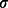 is always negative; hence any increase in 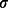 is detrimental to the population in this special case; this is consistent with prior work relating the effects of increased environmental variation to 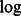-concavity of the response function [34]–[37] because for 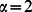, the response function is 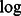-concave. Analysis of the absolute ratio of the two sensitivities, which is 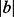, answers our second question: if mean and standard deviation are perturbed by the same amount, which causes the greater impact on the growth rate? For 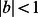, changes in 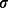 have a greater effect, whereas for 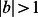, changes in mean environment are more important. For fixed values of 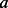 and 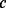, larger 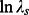 happens only through smaller 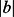 (recall 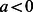), which means the absolute ratio of the two sensitivities is smaller; so larger long-term growth rates mean greater relative sensitivity of the growth rate to changes in environmental variability.

**Figure 2:**
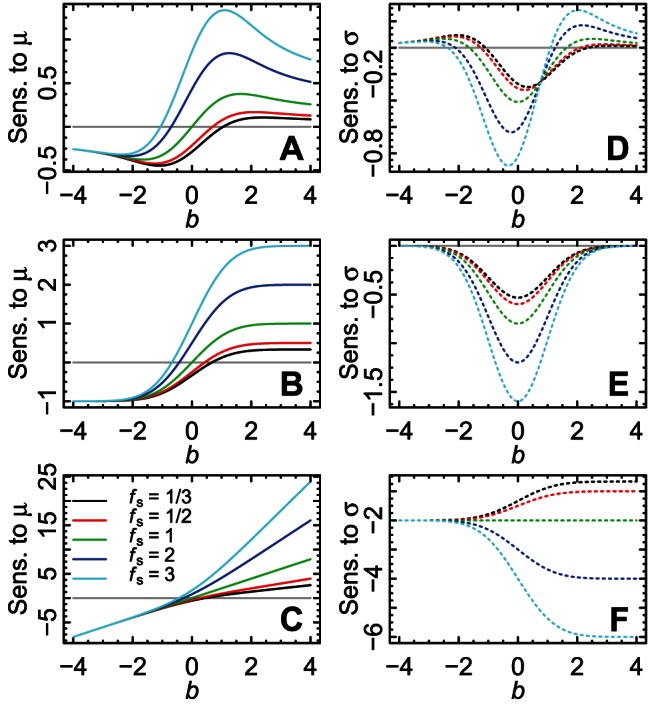
Comparison of signs of sensitivities. The sensitivities 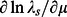 (A–C) and 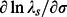 (D–F) are displayed; they were calculated numerically (Sections S3 and S4 in File S1). For 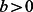, the slope of the response function at the mean value of the local environment is positive (e.g., Figure 1B), and for 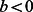, the slope is negative; therefore the sensitivity to changes in 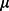 is largely of the same sign as that of the slope of the response function, as expected (A–C). The message here is that shifts of the mean environment toward the location of the peak of the response function usually cause an increase in 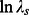, as expected, except possibly for some mean environments close to the peak. For 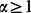, sensitivity to changes in 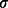 is always negative (E–F); for 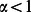, sensitivity to changes in 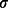 can be positive (D). The message here parallels prior work: for a log-concave response function (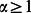), increased environmental variance always reduces the long-term stochastic growth rate; but for 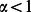, the reverse can be true (see main text). Sensitivities did not depend on 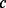. Signs of all sensitivities are identical to those displayed here for other values of 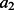 because changing 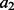 only rescales the vertical axes of all panels (Sections S3 and S4 in File S1). Here, 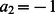, and 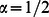 (A, D), 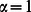 (B, E), or 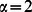 (C, F).

**Figure 3:**
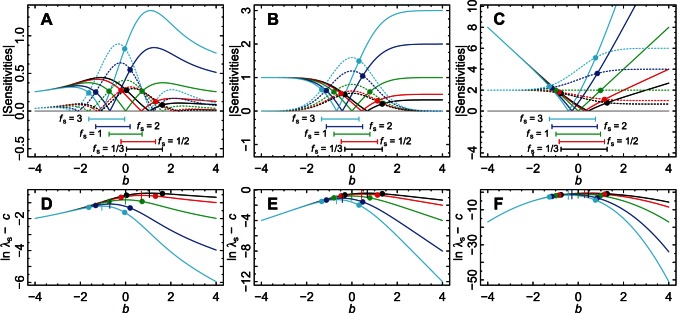
Comparison of the magnitudes of sensitivities. The relative sizes of 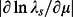 and 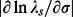 indicate which has a stronger effect on the long-term stochastic growth rate, changes in the mean of the environment (if 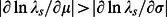) or changes in the standard deviation of the environment (if 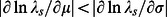); relative sizes of these quantities are depicted here. (A–C) Absolute values of the sensitivities of Figure 2; solid lines are 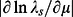 (taking 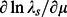 from Figure 2A–C) and dotted lines are 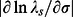 (taking 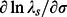 from Figure 2D–F). Dots indicate points at which solid and dotted lines of the same color cross, and hence where changes in mean environment become more important than changes in the standard deviation of the environment, or vice versa. Dots line up with the endpoints of the ranges below each plot and indicate the 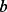 for which 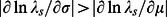 (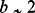). The message here is that when the mean environment is close to the peak of the response function, changes in the standard deviation of the environment have a bigger effect on the long-term stochastic growth rate than changes in the mean of the environment. (D–F) The difference 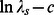, which shows how 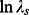 depends on 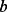. Dots are placed to line up with those in panels A–C, to show that 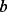 for which 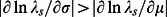 correspond to 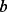 for which 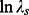 is large. Vertical lines indicate maxima. The maxima occur at values of 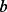 for which the sensitivity to changes in 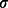 is greater than the sensitivity to changes in 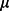. Here 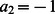, and 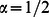 (A, D), 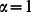 (B, E), or 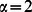 (C, F). Conclusions are identical to those displayed here for other values of 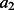 because changing 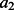 only rescales the vertical axes of all panels (Sections S3 and S4 in File S1).

Log response functions may often be asymmetric and 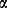 may differ from 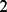, so how contingent are the above results on the assumptions made by the special case? We numerically analyzed the sensitivities of 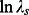 for a range of values of 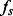 and for 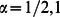 and 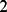 and results remain largely the same in substance. Figure 2A–C shows that 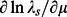, plotted against 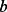, changes sign from negative to positive at a value of 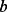 close to 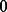, with some small variation in the value of 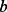 at which the sign changes, depending on the values of 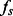 and 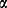. Figure 2D–F illustrates that for 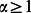, 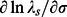 is always negative. For 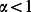, this sensitivity can be positive for larger values of 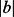. Since 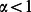 means parts of the 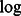-response function, 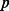, are convex, and earlier work shows that convexity of the 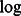-response function is associated with the possibility that increased environmental variance can benefit populations [34]–[37], the result from our model that 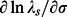 can be positive for 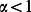 is consistent with earlier work. Figure 3 compares the absolute magnitudes of the sensitivities. For 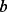 close to 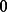, the sensitivity of 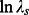 to changes in 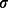 is generally comparable or larger in magnitude than the sensitivity to changes in 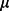. The specific interval of 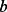 in which the sensitivity of 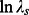 to changes in 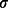 is larger varies depending on 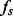 and 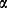. But regardless of this variation the conclusion holds that for small 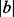 (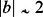 for the model parameters we examined), changes in environmental standard deviation are expected to be comparably or more important for long-term stochastic growth rate than changes of the same magnitude in the mean environment. This conclusion holds regardless of the concavity of 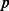, controlled by 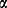. This suggests that the overwhelming emphasis of past research on the impacts on populations of changes in means of environmental states is misplaced and more attention should be paid to impacts of changes in environmental variability. Generality of the results to different distributions of 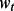 and different parameterizations of 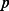 is explored in Section S7 in File S1. Figure 3D–F shows that for given 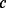 and 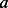, larger values of 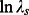 are within the range for which 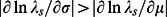, i.e., across a species environmental range, populations with comparatively higher growth rates are likely to be more affected by changes in variability of the environment than changes in mean.

### Results of Climate Data Analysis

**Figure 4:**
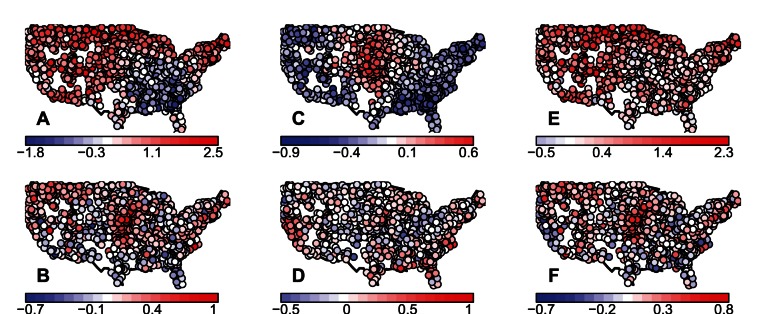
Relative changes in mean and standard deviation of climate variables in the United States. Both the mean and standard deviation of biologically important climate variables are changing; this figure indicates the relative magnitudes of these changes. If 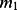 and 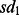 are the mean and standard deviation of winter temperature in a location for the period 1911–1945, and 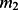 and 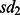 are the mean and standard deviation of winter temperature in the same location for the period 1976–2010, then: panel A shows 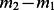, the degree of change in environmental mean; panel C shows 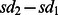, the degree of change in environmental standard deviation; and panel E shows 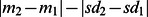, which indicates the relative magnitudes of these changes and is positive when changes in mean exceed changes in standard deviation and negative otherwise. White corresponds to no change on A–D and to equal changes in mean and standard deviation on E–F. Environmental variables depicted are winter mean temperature (A, C, E) and total spring precipitation (B, D, F). Mean and standard deviation of total spring precipitation (B, D, F) use the square root of the precipitation values (Methods). Other weather variables are shown in Figure S1 in Section S8 in File S1. The main message here is that changes in the means of most environmental variables are slightly but not markedly bigger than changes in their standard deviations.

The third question posed in the Introduction was: what are the relative magnitudes of observed changes in mean and standard deviation of climate variables? Results are shown for mean winter temperature and total spring precipitation in Figure 4, and for mean summer temperature, minimum winter temperature, and maximum summer temperature in Figure S1 in Section S8 in File S1. The magnitudes of changes in the means of all variables, except total spring precipitation, were generally slightly but not markedly larger than those of standard deviations. For total spring precipitation, changes in mean and standard deviation were of almost the same magnitude. Results are also spatially heterogeneous. The only variable for which changes in standard deviation are of the same sign throughout most of the United States is minimum winter temperature (Figure S1E in Section S8 in File S1), where variability decreased from 1911–1945 to 1976–2010. For all other variables, the sign and magnitude of changes depend on location. Changes in mean were generally slightly but not markedly bigger in magnitude than changes in standard deviation at local scales too (Figure 4E–F), although there are many locations and weather variables where the reverse is true (e.g., for summer mean temperature and precipitation). Although changes in means were more often larger than changes in standard deviation, both types of changes were similar in size, so results comparing relative sensitivities of long-term stochastic growth rate can also be interpreted as approximately reflecting the relative importance of the two types of environmental change for population dynamics. Results were very similar when linear detrending or no detrending were used in place of quadratic detrending.

## Discussion

We showed for a simple model how the effects on population dynamics of changes in the mean and variability of an environmental variable compare. Our results indicate that for small 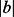, changes in the standard deviation of the environment are at least comparably important to changes in the mean environment. In other words, whenever the distribution of values of the local environment is close to the ideal environment, changes in environmental variability will be comparably or more important than changes in environmental mean for a population's growth rate. Recall that 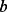 controls the extent to which the distribution of possible local environments deviates from the value of the environmental variable at which the response function peaks. We discuss the contrasting implications of these results for two different kinds of populations: those living close to or far from their ideal environment.

Populations living close to their ideal environment, such as those in the centre of the species environmental range, are interpreted in our model as those having small 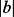. Populations of pests and insect disease vectors that live close to their ideal environment are of special interest because growth rates are highest and associated economic and health problems are worst in those locations reumanetal06, reumanetal08a, chavesetal11. Populations of exploited species, or of species that provide a major food supply for exploited species (e.g., copepods), are also of greatest interest, for economic reasons, in locations close to the species' ideal environments. For these populations, our results show that any increase in variability of the environment is detrimental, and that furthermore, changes in variability are more important than changes in mean. Given that the variability of temperature has decreased in many locations of the United States over the past 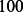 years, our model suggests that pests and disease vectors, but also potentially some exploited species, may stand to benefit from ongoing climate change in areas where the environment is already ideal for the species and they are already most prevalent.

Climate change has led to shifts and contractions in species' range sizes [2], [54] compounded by habitat loss and fragmentation [55]. Populations struggling to adapt to rapid climate changes will often be those living on the trailing edge of changing species ranges, where environmental conditions are suboptimal. Such populations may be of conservation interest; they are interpreted in our model as having large 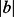. For these populations, environmental variability can be beneficial if the log response function is described by 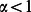, i.e., if it is convex. Our results also show that for these populations, changes in mean environmental conditions have a greater effect than changes in variability.

### Comparisons with other Studies

Prior studies, mentioned in the Introduction, have compared the impacts of changes in mean and variability of vital rates on 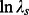, generally finding that populations are more sensitive to changes in vital rate means than they are to changes in vital rate standard deviations. Our study complements these earlier studies by using a response function to compare the impact of changes in mean and variability of the environment on 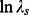. Our finding that changes in environmental variability can be more important than changes in environmental mean stands in counterpoint to the earlier results, and emphasizes the non-equivalence of studying the effects of changes in environmental and vital rate distributions. Morris *et al.* morrisetal08 concluded that although all species they examined were more sensitive to changes in vital rate means than variances, the greater importance of changes in means was reduced for shorter-lived, faster growing species. Our result that faster-growing populations, i.e., those close to their ideal environment, are more susceptible to changes in environmental variance appears to parallel the result of Morris *et al.*, but for environments instead of vital rates.

Only two empirical studies currently exist that can be directly compared to our theoretical predictions, and they provide support for our conclusions, though with caveats. Van de Pol *et al.* [37] and Jonzén *et al.* [41] used structured population models, parameterized for a population of oystercatchers [37] and a population of red kangaroos [41]. The oystercatcher population has been declining at a rate of 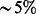 per year vandepoletal10; it therefore may be living in less than ideal conditions. Van de Pol *et al.* conclude, as our model would suggest, that changes in mean environmental conditions will have a greater effect on this population than do changes in variability. The red kangaroo population of [41] probably lives in a closer-to-ideal environment for the species, as it has a substantially positive 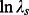: Jonzén *et al.* estimate that growth rate will be greater than 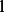 even with annual harvesting of up to 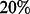. Consistent with our model, the sensitivity of 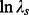 to changes in mean rainfall (after converting the elasticities provided in [41] to sensitivities) is only 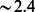 times greater in magnitude than that to changes in the standard deviation of rainfall: sensitivity to changes in standard deviation is important for the kangaroo population. These comparisons are subject to the caveats that: 1) other hypotheses besides a sub-optimal environment have been proposed as possible causes of the decline of the oystercatcher population [56]; 2) only these two studies are currently available for comparison. More insight can be gained in future work by replicating the efforts of [37] and [41] for other populations. This is a non-trivial effort. Many years worth of data are necessary for each population (e.g., 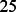 years of data were used in [37]). Each monitored population would correspond to a single point in parameter space of a general theoretical analysis. A principle value of our modeling is in guiding future empirical work. Our findings help inform what populations may be of interest to compare. We suggest the comparison of populations thought to be living in close-to-ideal conditions with those far from ideal conditions. For example, one could replicate the study of van de Pol *et al.* with other oystercatcher populations across a gradient of environmental conditions, including expanding populations.

The distinction between 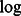-concave and 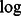-convex response functions has been emphasized in prior work as important for whether increased environmental variance will increase or decrease population long-term stochastic growth rate [34]–[37]; for 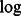-concave (respectively, 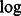-convex) response functions, it is easy to see that geometric-mean vital rate values are lower (respectively, higher) under increased environmental variance. However, if the mean local environment maximizes or nearly maximizes a response function, then the response function is effectively 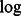-concave for relevant environments: increased environmental variance can only decrease geometric-mean vital rate values, because increased variance includes more environments that are farther from the environment that optimizes the vital rate. Thus, distance of the mean local environment from the population ideal environment (with distance measured in units of the standard deviation of the local environment) supersedes the question of 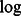-concavity. The 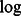-concavity distinction still makes a difference far from the ideal environment (compare figures 2D–F).

### Biological Realism and Possible Future Work

Our model is simple, but main conclusions are intuitively sensible and seem likely to generalize to other models. Because the relationship between log annual population growth rate and environment peaks in our model at the optimum environment, geometric-mean growth rate will not be strongly sensitive to changes in environmental mean when environmental mean is close to optimal. For instance, when 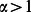, the slope of the 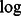-response function close to the ideal environment is close to zero, so small changes in mean environment from the optimum have little effect on geometric-mean annual growth rate. On the other hand, because rates decline with departures from the optimal environment in either direction of the optimum, changes in environmental variance may strongly affect geometric-mean annual growth when the mean environment is optimal, because larger environmental variances include more values of the environment that are far from the optimum. This simple conceptual reasoning is made precise by our modeling results. Similar reasoning holds for any model for which all vital rates can be written approximately as functions of a single environmental variable. For any fixed value of environmental variance, 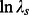 must have a maximum at some value of the environmental mean. Sensitivity of 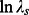 to changes in the mean environment must approach zero here, as long as 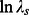 is a smooth function of environmental mean. For fixed environmental variance, the ideal mean environment is the one that maximizes 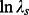. As long as the local environment is close to this ideal environment, one therefore expects sensitivity of 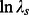 to changes in the mean environment to be very small, and hence it is likely that sensitivity will be greater to changes in the standard deviation of the environment. This reasoning applies to stage structured models, and to density-dependent models if 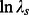 is replaced with some other measure of population success (e.g., average population size). The potential importance of these observations for real populations seems largely overlooked by prior work, which generally compares the importance of changes in the means and variances of vital rates. For populations strongly affected by two or more environmental variables, potentially acting on different vital rates, there may be no single ideal environment. Instead, tradeoffs may occur, whereby various mean values for one environmental variable can be paired with different mean values of the other variable to maximize 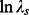. This may be an important topic for future study.

Many species show a ''storage effect,'' a well-studied phenomenon by which some life stages are less susceptible to adverse environments than other life stages; storage effects are a classic mechanism of species coexistence [57]–[59]. Our model cannot incorporate storage effects because it is unstructured, so investigating how storage effects impact the main conclusions of this study may be another important topic for future research. Species exhibiting storage effects include long-lived species with resistant adult stages (e.g., trees), as well as species with spores or seed banks (e.g., fungi and annual grasses; [60]). Our model corresponds instead to another large category of species with no storage stage, e.g., insects and other organisms that overwinter as eggs which are not viable beyond the following spring. Although eggs may be insensitive to the winter environment, this is not a storage stage as long as eggs cannot remain viable beyond spring. The insensitivity of storage phases to bad environments may make it appear as though species with storage phases must be more sensitive to changes in environmental means than to changes in environmental variation. However, storage phases are only insensitive to environmental variation in the sense that they can tolerate bad conditions. From another perspective, storage phases are very sensitive to environmental variation because they respond strongly to good environments. For instance, spores or seeds in a seed bank emerge when conditions are suitable. Also, adult stages may reproduce prolifically under good conditions. This alternative form of sensitivity to the environment may translate into sensitivity of population long-term stochastic growth rates to changes in environmental variability. Both modified standard deviation of environment and changes in the mean of the environment can decrease the fraction of years for which environmental conditions are acceptable for seeds or other storage phases to become active. If all vital rates are affected primarily by the same environmental variable, then the logic of the prior paragraph still applies, even if there are storage effects, suggesting the main conclusions of this study may still hold in many cases even with storage effects.

Stage structure must be introduced into the model to analyze storage effects or to illuminate possible consequences of multiple environmental variables acting on different vital rates. For a general stage-structured model, 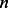 vital rates or stochastic matrix elements would be linked to 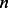 potentially different environmental variables 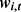 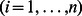 by different response functions, each with its own 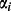, 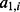, 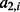, 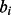, and 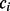, resulting in 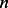 sensitivities of 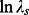 to changes in 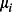 and 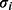. The 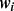 may also be correlated and this correlation structure may be affected in unknown ways by climate change. The mathematical complexity here may be difficult to manage in the general case. Not all parameter combinations are equally likely, though. For instance, slow-growing populations such as oystercatchers have high adult survival rates probably described by a concave function, and have low fecundity rates likely described by a convex function [37]. A similar pattern is observed in many organisms (e.g., fish [61]; perennial and annual plants [62], [63]). Whether these biological regularities can be formalized and used to simplify the mathematics remains to be seen. If a general model proves too complicated to immediately provide insight, a sensible next step may be a 2×2 matrix model of a population with juveniles and adults (non-semelparous, as semelparous populations are covered by our model; Section S9 in File S1). Such a model would make it possible to study the differing impacts of climate change on fecundity and survival rates, as well as effects that may only emerge when some stage structure is present. For an age or stage structured model, the exact formulation of 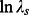 used in this study would no longer be valid, but Tuljapurkar's [45], [48] approximation could be used. For the unstructured case, the approximation yields qualitatively similar results to the ones presented here (results not shown). An alternative approach would be possible if sufficiently many case studies were available for which population models were empirically established, with vital rates explicitly linked to environmental variables. Given such a model, it is straightforward to evaluate the relative sensitivities of 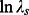 to changes in the mean and standard deviation of the environmental variable, as done in references [37] and [41], but a substantial number of case studies would be needed to draw general conclusions.

The long-term stochastic growth rate for a stage-structured model is also affected by autocorrelation in the environment [42], [45], [48]. The autocorrelation of environmental variables is also changing due to climate change [64]. It would be possible, using a stage-structured model, to compare the relative effects of changes in mean, variance, and autocorrelation of the environment on population dynamics (as done for a single oystercatcher population in [65]). Finally, the sensitivities of 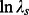 are linear approximations of the functions that relate 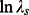 to 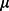 and 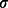, and therefore assume small changes in the environment. More substantial environmental changes may entail nonlinearities for which a linear approximation is no longer sufficient. An examination of such nonlinear effects may be analytically intractable, though simulations and numeric work may provide insights.

We considered annual environmental variables because most demographic data and models of the type we consider have an annual time step. But annual environmental variables, such as spring mean temperature, are averages of shorter-time-scale events (e.g., spring mean temperature may be calculated as the mean of daily temperatures during spring). We do not here consider standard deviation of, for instance, daily temperatures measured in the spring, nor do we consider the effects of changes in such a standard deviation. Other studies do consider these shorter time-scales [66] instead of considering inter-annual standard deviations, as we do. A comparison of the importance of changes in inter- and intra-annual standard deviation may be an interesting topic of future research.

Common sense and appropriate empirical evidence support the assumption of a peaked, skewed response function, but we admit the possibility that other response functions could occur in some circumstances; our analytic approach could easily be adapted to essentially any response function. Focal-population studies, such as [67], in which vital rates of a single population are related to values of an environmental variable experienced by that population, need not necessarily show a peak in the response function, even when one exists. A population would need to be living close to its ideal environment for the peak in the response function to be apparent in locally gathered data, and even in that case, unless the local environmental variability were large, the response function may appear to be flat to within the accuracy of measurement of vital rates or annual growth rates. Local environmental variables do not usually span much of the range of environmental values the species could potentially experience across its geographic range, hence peaks will often not be visible in such studies. This does not, however, preclude the presence of a peak in the whole response function, but instead indicates that many studies look at narrow environmental ranges [29]. Different kinds of studies in which response functions are measured across a wider range of values of the environmental variable are more appropriate for assessing the shape of a response function. Empirical evidence of peaked response functions in both ectotherms and endotherms can be found in [27]–[29], [31], [32], and theoretical support is provided by [30] (some results of these studies are summarized in Section S7). Apparently saturating response functions are usually more likely to be unimodal response functions, with a peak that is remote from the range measured in a locally focussed study. Threshold response functions may also be possible, for instance if populations respond differently below and above the freezing point of water. However, these seem more likely to be important at shorter time-scales (e.g., daily or hourly) than the annual time-scales considered here. The annualized environmental variables we use are more likely to be statistically related to annually measured vital rates, and will not usually have discontinuous thresholds. Nevertheless, our analytic approach can easily be applied to any alternative response function if a particular shape not encompassed by the parameterization we have used is found to common enough to warrant study (Section S7).

## Supporting Information

File S1 **Supporting information.**
(PDF)

## References

[1] IPCC (2007) Climate Change 2007: Synthesis Report. Contribution of Working Groups I, II and III to the Fourth Assessment Report of the Intergovernmental Panel on Climate Change. Geneva, Switzerland: IPCC.

[2] Parmesan C, Ryrholm N, Stefanescu C, Hill JK, Thomas CD, et al (1999) Poleward shifts in geographical ranges of buttery species associated with regional warming. Nature 399(6736): 579–583 doi:0.1038/21181.

[3] Morris WF, Pfister CA, Tuljapurkar S, Haridas CV, Boggs CL, et al (2008) Longevity can buffer plant and animal populations against changing climatic variability. *Ecology* 89(1): 19–25 doi: 10.1890/07-0774.1. 18376542 10.1890/07-0774.1

[4] Boer GJ (2010) Changes in interannual variability and decadal potential predictability under global warming. *J Climate* 22: 3098–3109 doi:10.1175/2008JCLI2835.1.

[5] Schoener TW, Spiller DA (1992) Is extinction rate related to temporal variability in population size? An empirical answer for orb spiders. *Am Nat* 139(6): 1176–1207 doi:10.1086/285381.

[6] McLaughlin JF, Hellmann JJ, Boggs CL, Ehrlich PR (2002) Climate change hastens population extinctions. *Proc Natl Acad Sci* U S A 99(9): 6070–6074 doi:10.1073/pnas.052131199. 11972020 10.1073/pnas.052131199 PMC122903

[7] Tuljapurkar S, Horvitz CC, Pascarella JB (2003) The many growth rates and elasticities of populations in random environments. *Am Nat* 162(4): 489–502 doi:10.1086/378648. 14582010 10.1086/378648

[8] Tews J, Jeltsch F (2004) Modelling the impact of climate change on woody plant population dynamics in South African savanna. *BMC Ecol* 4(1): 17 doi:10.1186/1472-6785-4-17. 15606921 10.1186/1472-6785-4-17 PMC544358

[9] Chaves LF, Morrison AC, Kitron UD, Scott TW (2011) Nonlinear impacts of climatic variability on the density-dependent regulation of an insect vector of disease. *Glob Change Biol* 18(2): 457–468 doi:10.1111/j.1365-2486.2011.02522.x.

[10] Karl TR, Knight RW, Plummer N (1995) Trends in high-frequency climate variability in the twentieth century. *Nature* 377: 217–220 doi:10.1038/377217a0.

[11] R?ais?anen J (2002) CO2-induced changes in interannual temperature and precipitation variability in 19 CMIP2 experiments. *J Climate* 15(17): 2395–2411 doi:10.1175/1520-0442(2002)015.

[12] Sun F, Roderick ML, Farquhar GD, Lim WH, Zhang Y, et al (2010) Partitioning the variance between space and time. *Geophys Res Lett* 37(12): L12704 doi:10.1029/2010GL043323.

[13] Parker DE, Folland CK, Bevan A, Jones PD (1994) Interdecadal changes of surface temperature since the late nineteenth century. *J Geophys Res* 99(D7): 14373–14399 doi:10.1029/94JD00548.

[14] Vinnikov KY, Robock A (2002) Trends in moments of climatic indices. *Geophys Res Lett* 29(2): 14–1–14-4 doi:10.1029/2001GL014025.

[15] Michaels PJ, Bailing RC, Vose RS, Knappenberger PC (1998) Analysis of trends in the variability of daily and monthly historical temperature measurements. *Clim Res* 10: 27–33 doi: 10.3354/cr010027.

[16] Svoma BM, Balling RC (2010) United States' interannual precipitation variability over the past century: is variability increasing as predicted by models? *Phys Geogr* 31(4): 307–318 doi: 10.2747/0272-3646.31.4.307.

[17] Hansen J, Sato M, Ruedy R (2012) Perception of climate change. Proc *Natl Acad Sci* USA 109(37): E2415–E2423 doi:10.1073/pnas.1205276109. 22869707 10.1073/pnas.1205276109 PMC3443154

[18] Hunt BG, Elliott TI (2004) Interaction of climatic variability with climatic change. *Atmos Ocean* 42(3): 145–172 doi:10.3137/ao.420301.

[19] Stouffer RJ, Wetherald RT (2007) Changes of variability in response to increasing greenhouse gases. Part I: Temperature. *J Climate* 20(21): 5455–5467 doi:10.1175/2007JCLI1384.1.

[20] Sakai D, Itoh H, Yukimoto S (2009) Changes in the interannual surface air temperature variability in the Northern Hemisphere in response to global warming. *J Meteor Soc Japan* 87(4): 721–737 doi:10.2151/jmsj.87.721.

[21] Laakso J, Kaitala V, Ranta E (2001) How does environmental variation translate into biological processes? *Oikos* 92: 119–122 doi:10.1034/j.1600-0706.2001.920114.x.

[22] Atkinson D, Sibly RM (1997) Why are organisms usually bigger in colder environments? Making sense of a life history puzzle. *Trends Ecol Evol* 12(6): 235–239 doi:10.1016/S0169-5347(97)01058-6. 21238056 10.1016/s0169-5347(97)01058-6

[23] Huey RB, Stevenson RD (1979) Integrating thermal physiology and ecology of ectotherms: a discussion of approaches. *Am Zool* 19(1): 357–366 doi:10.1093/icb/19.1.357.

[24] Savage VM, Gillooly JF, Brown JH, West GB, Charnov EL (2004) Effects of body size and temperature on population growth. *Am Nat* 163(3): 429–441 doi:10.1086/381872. 15026978 10.1086/381872

[25] Forster J, Hirst AG, Woodward G (2011) Growth and development rates have different thermal responses. *Am Nat* 178(5): 668–678 doi:10.1086/662174. 22030735 10.1086/662174

[26] Begon M, Harper JL, Townsend CR (1996) Ecology: Individuals, Populations and Communities. Oxford, UK: Blackwell Science Ltd.

[27] Huey RB, Berrigan D (2001) Temperature, demography, and ectotherm fitness. *Am Nat* 158(2): 204–210 doi:10.1086/321314. 18707349 10.1086/321314

[28] Deutsch CA, Tewksbury JJ, Huey RB, Sheldon KS, Ghalambor CK, et al (2008) Impacts of climate warming on terrestrial ectotherms across latitude. *Proc Natl Acad Sci* U S A 105(18): 6668–6672 doi:10.1073/pnas.0709472105. 18458348 10.1073/pnas.0709472105 PMC2373333

[29] Dell AI, Pawar S, Savage VM (2011) Systematic variation in the temperature dependence of physiological and ecological traits. *Proc Natl Acad Sci* U S A 108(26): 10591–10596 doi: 10.1073/pnas.1015178108. 21606358 10.1073/pnas.1015178108 PMC3127911

[30] Amarasekare P, Savage V (2012) A framework for elucidating the temperature dependence of fitness. *Am Nat* 179(2): 178–191 doi:10.1086/663677. 22218308 10.1086/663677

[31] Jenouvrier S, Holland M, Stroeve J, Barbraud C, Weimerskirch H, et al (2012) Effects of climate change on an emperor penguin population: analysis of coupled demographic and climate models. *Glob Change Biol* 18(9): 2756–2770 doi:10.1111/j.1365-2486.2012.02744.x. 10.1111/j.1365-2486.2012.02744.x 24501054

[32] Thomas MK, Kremer CT, Klausmeier CA, Litchman E (2012) A global pattern of thermal adaptation in marine phytoplankton. *Science* 338(6110): 1085–1088 doi:10.1126/science.1224836. 23112294 10.1126/science.1224836

[33] Boyles JG, Seebacher F, Smit B, McKechnie AE (2011) Adaptive thermoregulation in endotherms may alter responses to climate change. *Integr Comp Biol* 51(5): 676–690 doi:10.1093/icb/icr053. 21690108 10.1093/icb/icr053

[34] Ruel JJ, Ayres MP (1999) Jensen's inequality predicts effects of environmental variation. *Trends Ecol Evol* 14(9): 361–366 doi:10.1016/S0169-5347(99)01664-X. 10441312 10.1016/s0169-5347(99)01664-x

[35] Drake JM (2005) Population effects of increased climate variation. *Proc R Soc Lond B Biol Sci* 272(1574): 1823–1827 doi:10.1098/rspb.2005.3148. 10.1098/rspb.2005.3148 PMC1559868 16096095

[36] Boyce MS, Haridas CV, Lee CT (2006) The NCEAS Stochastic Demography Working Group (2006) Demography in an increasingly variable world. *Trends Ecol Evol* 21(3): 141–148 doi: 10.1016/j.tree.2005.11.018. 16701490 10.1016/j.tree.2005.11.018

[37] van de Pol M, Vindenes Y, Sæther BE, Engen S, Ens BJ, et al (2010) Effects of climate change and variability on population dynamics in a long-lived shorebird. *Ecology* 91(4): 1192–1204 doi: 10.1890/09-0410.1. 20462133 10.1890/09-0410.1

[38] Haridas CV, Tuljapurkar S (2005) Elasticities in variable environments: properties and implications. *Am Nat* 166(4): 481–495 doi:10.1086/444444. 16224704 10.1086/444444

[39] Ezard THG, Coulson T (2010) How sensitive are elasticities of long-run stochastic growth to how environmental variability is modelled? *Ecol Model* 221(2): 191–200 doi: 10.1016/j.ecolmodel.2009.09.017.

[40] Coulson T, MacNulty DR, Stahler DR, vonHoldt B, Wayne RK, et al (2011) Modeling effects of environmental change on wolf population dynamics, trait evolution, and life history. *Science* 334(6060): 1275–1278 doi:10.1126/science.1209441. 22144626 10.1126/science.1209441

[41] Jonz_en N, Pople T, Knape J, Sk?old M (2010) Stochastic demography and population dynamics in the red kangaroo Macropus rufus. *J Anim Ecol* 79(1): 109–116 doi:10.1111/j.1365-2656.2009.01601.x. 19674179 10.1111/j.1365-2656.2009.01601.x

[42] Caswell H (2001) Matrix Population Models: Construction, Analysis, and Interpretation. Sunderland, MA: Sinauer Associates, Inc.

[43] Morris WF, Doak DF (2002) Quantitative Conservation Biology: Theory and Practice of Population Viability Analysis. Sunderland, Massachusetts: Sinauer Associates, Inc.

[44] Lewontin RC, Cohen D (1969) On population growth in a randomly varying environment. *Proc Natl Acad Sci* U S A 62(4): 1056–1060 doi:10.1073/pnas.62.4.1056. 5256406 10.1073/pnas.62.4.1056 PMC223613

[45] Tuljapurkar SD (1982) Population dynamics in variable environments. III. Evolutionary dynamics of r -selection. *Theor Popul Biol* 21(1): 141–165 doi:10.1016/0040-5809(82)90010-7.

[46] Lande R, Orzack SH (1988) Extinction dynamics of age-structured populations in a uctuating environment. *Proc Natl Acad Sci* U S A 85(19): 7418–7421 doi:10.1073/pnas.85.19.7418. 3174643 10.1073/pnas.85.19.7418 PMC282198

[47] Lande R, Engen S, Sæther BE (2003) Stochastic Population Dynamics in Ecology and Conservation. Oxford Series in Ecology and Evolution. Oxford, UK: Oxford University Press.

[48] Tuljapurkar SD (1990) Population Dynamics in Variable Environments. New York, NY: Springer-Verlag.

[49] Ezard THG, Gaillard JM, Crawley MJ, Coulson T (2008) Habitat Dependence and Correlations between Elasticities of Long-Term Growth Rates. *Am Nat* 172(3): 424–430 doi:10.1086/589897. 18637759 10.1086/589897

[50] Menne MJ, Williams Jr CN, Vose RS (2009) The U.S. Historical Climatology Network monthly temperature data, version 2. *Bull Amer Meteor Soc* 90(7): 993–1007 doi:10.1175/2008BAMS2613.1.

[51] National Climatic Data Center, National Oceanic and Atmospheric Administration (2011). The USHCN Version 2 Serial Monthly Dataset. Available: ftp://ftp.ncdc.noaa.gov/pub/data/ushcn/v2/monthly/. Accessed 2011 April 16.

[52] Reuman DC, Desharnais RA, Costantino RF, Ahmad OS, Cohen JE (2006) Power spectra reveal the inuence of stochasticity on nonlinear population dynamics. Proc *Natl Acad Sci* U S A 103(49): 18860–18865 doi:10.1073/pnas.0608571103. 17116860 10.1073/pnas.0608571103 PMC1693752

[53] Reuman DC, Costantino RF, Desharnais RA, Cohen JE (2008) Colour of environmental noise affects the nonlinear dynamics of cycling, stage-structured populations. *Ecol Lett* 11: 820–830 doi:10.1111/j.1461-0248.2008.01194.x. 18479454 10.1111/j.1461-0248.2008.01194.x

[54] Thomas CD, Lennon JJ (1999) Birds extend their ranges northwards. *Nature* 399(6733): 213–213 doi:10.1038/20335.

[55] Sala OE, Chapin III SF, Armesto JJ, Berlow E, Bloomfield J, et al (2000) Global biodiversity scenarios for the year 2100. Science 287(5459): 1770–1774 doi:10.1126/science.287.5459.1770 10710299 10.1126/science.287.5459.1770

[56] van de Pol M (2006) State-dependent life history strategies: a long-term study on Oystercathers. Ph.D. thesis, University of Groningen.

[57] Levins R (1979) Coexistence in a variable environment. *Am Nat* 114(6): 765–783.

[58] Armstrong RA, McGehee R (1980) Competitive exclusion. *Am Nat* 115(2): 151–170 doi: 10.1086/283553.

[59] Chesson PL (1986) Community Ecology, chapter Environmental variation and the coexistence of species. New York, USA: Harper & Row, 240–256.

[60] Hairston NG, Ellner S, Kearns CM (1996) Population Dynamics in Ecological Space and Time, chapter Overlapping generations: The storage effect and the maintenance of biotic diversity. Chicago, USA: The University of Chicago Press, 109–146.

[61] Gulland JA (1982) Why do fish numbers vary? *J Theor Biol* 7(1): 69–75 doi:10.1016/0022-5193(82)90277-6.

[62] Grubb PJ (1977) The maintenance of species-richness in plant communities: the importance of the regeneration niche. *Biol Rev* 52(1): 107–145 doi:10.1111/j.1469-185X.1977.tb01347.x.

[63] Facelli JM, Chesson PL, Barnes N (2005) Differences in seed biology of annual plants in arid lands: a key ingredient of the storage effect. *Ecology* 86: 2998–3006 doi:10.1890/05-0304.

[64] García-Carreras B, Reuman DC (2011) An empirical link between the spectral colour of climate and the spectral colour of field populations in the context of climate change. *J Anim Ecol* 80(5): 1042–1048 doi:10.1111/j.1365-2656.2011.01833.x. 21466552 10.1111/j.1365-2656.2011.01833.x

[65] van de Pol M, Vindenes Y, Sæther BE, Engen S, Ens BJ, et al (2011) Poor environmental tracking can make extinction risk insensitive to the colour of environmental noise. *Proc R Soc Lond B Biol Sci* 278(1725): 3713–3722 doi:10.1098/rspb.2011.0487. 10.1098/rspb.2011.0487 PMC3203500 21561978

[66] Campbell RD, Nouvellet P, Newman C, Macdonald DW, Rosell F (2012) The inuence of mean climate trends and climate variance on beaver survival and recruitment dynamics. *Glob Change Biol* 18(9): 2730–2742 doi:10.1111/j.1365-2486.2012.02739.x. 10.1111/j.1365-2486.2012.02739.x 24501052

[67] Coulson T, Catchpole EA, Albon SD, Morgan BJT, Pemberton JM, et al (2001) Age, sex, density, winter weather, and population crashes in Soay sheep. *Science* 292(5521): 1528 doi: 10.1126/science.292.5521.1528. 11375487 10.1126/science.292.5521.1528

