# Supplementary material for: Are Changes in the Mean or Variability of Climate Signals More Important for Long-Term Stochastic Growth Rate?
Source: PLoS One. 2013 May 14;8(5):e63974. doi: 10.1371/journal.pone.0063974 (PMC3653831; doi:10.1371/journal.pone.0063974)
Supplement: File S1 — Supporting information. (PDF) [file pone.0063974.s001.pdf]

Supporting Information

Section S1 Transforming the environmental variable

Let $\tilde{w}_t$ be the original, untransformed environmental variable. If X is any random variable with smooth cumulative distribution function (cdf) ϕ_X, and if ϕ_n is the standard normal cdf, then $\phi_n^{-1}(\phi_X(X))$ is standard normally distributed. This is a standard result. We assume $\tilde{w}_t$ is not irregularly distributed, so that it can be transformed to be normal in this way without violating the assumption that the response function, f, is single-peaked. If $\tilde{w}_t$ has been transformed to be normally distributed, then let

$$w_t = \alpha\,\tilde{w}_t + \beta$$

where $\alpha = 1/\mathrm{sd}(\tilde{w}_t)$ and $\beta = -\mathrm{E}(\tilde{w}_t)/\mathrm{sd}(\tilde{w}_t)$. This is standard-normally distributed.

Section S2 Derivation of $\ln \lambda_s$

Let $g(w_t) = p(w_t) - c$. Then,

$$\ln \lambda_s = \int_{-\infty}^{\infty} g(w_t)\,\varphi(w_t|\mu,\sigma)\,\mathrm{d}w_t + c \tag{1}$$

$$= a_1 \int_{-\infty}^{b} (-w_t + b)^{\alpha}\,\varphi(w_t|\mu,\sigma)\,\mathrm{d}w_t + a_2 \int_{b}^{\infty} (w_t - b)^{\alpha}\,\varphi(w_t|\mu,\sigma)\,\mathrm{d}w_t + c. \tag{2}$$

Therefore $\ln \lambda_s$ is a function of a_1, a_2, b, c, and α, i.e. the parameters that define the log response function.

Section S3 Derivation of $\partial \ln \lambda_s/\partial \mu$

Take the partial derivative of $\ln \lambda_s$ with respect to μ:

$$\frac{\partial \ln \lambda_s}{\partial \mu} = \int_{-\infty}^{\infty} p(w_t)\,\frac{\partial}{\partial \mu}\,\varphi(w_t|\mu,\sigma)\,\mathrm{d}w_t, \tag{3}$$

$$\frac{\partial}{\partial \mu}\varphi(w_t|\mu,\sigma) = \frac{\partial}{\partial \mu}\left[\frac{1}{\sigma\sqrt{2\,\pi}}\,\exp\!\left(\frac{-(w_t-\mu)^2}{2\,\sigma^2}\right)\right] \tag{4}$$

$$= \left(\frac{w_t - \mu}{\sigma^2}\right)\varphi(w_t|\mu,\sigma). \tag{5}$$

So

$$\frac{\partial \ln \lambda_s}{\partial \mu} = \int_{-\infty}^{\infty} p(w_t)\left(\frac{w_t - \mu}{\sigma^2}\right)\varphi(w_t|\mu,\sigma)\,\mathrm{d}w_t, \tag{6}$$

and

$$\left.\frac{\partial \ln \lambda_s}{\partial \mu}\right|_{\substack{\mu=0\\ \sigma=1}} = \int_{-\infty}^{\infty} g(w_t)\, w_t\, \varphi(w_t|\mu=0,\sigma=1)\,\mathrm{d}w_t + c\int_{-\infty}^{\infty} w_t\,\varphi(w_t|\mu=0,\sigma=1)\,\mathrm{d}w_t. \tag{7}$$

We need two lemmas to proceed.

Lemma S3.1

$$\int_0^{\infty} x^n\, e^{-\beta\, x^2}\,\mathrm{d}x = \frac{\Gamma(\gamma)}{2\,\beta^{\gamma}},$$

where $\Gamma(z)$ *is the Gamma function [1, pg. 255],* $\gamma = \frac{n+1}{2}$, $\mathrm{Re}\,\beta > 0$, *and* $\mathrm{Re}\,n > 0$.

Proof See [2, 3.326, pg. 337]. □

Lemma S3.2

$$\int_{-\infty}^{\infty} x^n\,\varphi_{0,1}(x)\,\mathrm{d}x = \begin{cases} \dfrac{\Gamma\left(\frac{n+1}{2}\right)2^{\frac{n}{2}}}{\sqrt{\pi}} & \textit{if } n \geq 0 \textit{ is an even integer}\\ 0 & \textit{if } n \geq 0 \textit{ is an odd integer.}\end{cases}$$

Proof

$$\int_{-\infty}^{\infty} x^n\,\varphi_{0,1}(x)\,\mathrm{d}x = \frac{1}{\sqrt{2\,\pi}}\int_{-\infty}^{\infty} x^n\,\exp\left(-\frac{x^2}{2}\right)\mathrm{d}x \tag{8}$$

$$= \begin{cases} \dfrac{2}{\sqrt{2\,\pi}}\displaystyle\int_0^{\infty} x^n\,\exp\left(-\frac{x^2}{2}\right)\mathrm{d}x & \text{if } n \text{ is even,}\\ 0 & \text{if } n \text{ is odd,}\end{cases} \tag{9}$$

$$= \begin{cases} \dfrac{2}{\sqrt{2\,\pi}}\,\dfrac{\Gamma\left(\frac{n+1}{2}\right)}{2\left(\frac{1}{2}\right)^{\frac{n+1}{2}}} & \text{if } n \text{ is even,}\\ 0 & \text{if } n \text{ is odd,}\end{cases} \tag{10}$$

$$= \begin{cases} \dfrac{\Gamma\left(\frac{n+1}{2}\right)2^{\frac{n}{2}}}{\sqrt{\pi}} & \text{if } n \text{ is even,}\\ 0 & \text{if } n \text{ is odd.}\end{cases} \tag{11}$$

□

Given lemma S3.2, the second term of equation (7) is equal to zero, so

$$\left.\frac{\partial \ln \lambda_s}{\partial \mu}\right|_{\substack{\mu=0\\ \sigma=1}} = a_2\left[f_s\int_{-\infty}^{b}(-w_t+b)^{\alpha}\,w_t\,\varphi(w_t|\mu=0,\sigma=1)\,\mathrm{d}w_t + \int_b^{\infty}(w_t-b)^{\alpha}\,w_t\,\varphi(w_t|\mu=0,\sigma=1)\,\mathrm{d}w_t\right]. \tag{12}$$

The sensitivity of $\ln \lambda_s$ to changes in μ therefore depends on a_2, f_s, b, and α. Some thoughts on how results can extend to other parameterizations of w_t and p are in Section S7.

Section S4 Derivation of $\partial \ln \lambda_s / \partial \sigma$

Take the partial derivative of $\ln \lambda_s$ with respect to σ:

$$\frac{\partial \ln \lambda_s}{\partial \sigma} = \int_{-\infty}^{\infty} p(w_t)\, \frac{\partial}{\partial \sigma}\, \varphi(w_t|\mu,\sigma)\, \mathrm{d}w_t, \tag{13}$$

$$\frac{\partial}{\partial \sigma}\varphi(w_t|\mu,\sigma) = \frac{\partial}{\partial \sigma}\left[\frac{1}{\sigma\sqrt{2\,\pi}}\, \exp\!\left(\frac{-(w_t-\mu)^2}{2\,\sigma^2}\right)\right] \tag{14}$$

$$= \frac{-1}{\sigma^2\sqrt{2\,\pi}}\, \exp\!\left(\frac{-(w_t-\mu)^2}{2\,\sigma^2}\right) + \frac{1}{\sigma\sqrt{2\,\pi}}\, \exp\!\left(\frac{-(w_t-\mu)^2}{2\,\sigma^2}\right)\left(\frac{(w_t-\mu)^2}{\sigma^3}\right) \tag{15}$$

$$= -\frac{1}{\sigma}\, \varphi(w_t|\mu,\sigma) + \frac{(w_t-\mu)^2}{\sigma^3}\, \varphi(w_t|\mu,\sigma). \tag{16}$$

Therefore

$$\frac{\partial \ln \lambda_s}{\partial \sigma} = \int_{-\infty}^{\infty} p(w_t)\left[-\frac{1}{\sigma}\, \varphi(w_t|\mu,\sigma) + \frac{(w_t-\mu)^2}{\sigma^3}\, \varphi(w_t|\mu,\sigma)\right] \mathrm{d}w_t, \tag{17}$$

and

$$\left.\frac{\partial \ln \lambda_s}{\partial \sigma}\right|_{\substack{\mu=0\\ \sigma=1}} = \int_{-\infty}^{\infty} (g(w_t)+c)\,(w_t^2-1)\,\varphi(w_t|\mu=0,\sigma=1)\,\mathrm{d}w_t \tag{18}$$

$$= \int_{-\infty}^{\infty} g(w_t)\,(w_t^2-1)\,\varphi(w_t|\mu=0,\sigma=1)\,\mathrm{d}w_t + c\int_{-\infty}^{\infty} w_t^2\, \varphi(w_t|\mu=0,\sigma=1)\,\mathrm{d}w_t - c. \tag{19}$$

Given lemma (S3.2),

$$\left.\frac{\partial \ln \lambda_s}{\partial \sigma}\right|_{\substack{\mu=0\\ \sigma=1}} = \int_{-\infty}^{\infty} g(w_t)\,(w_t^2-1)\,\varphi(w_t|\mu=0,\sigma=1)\,\mathrm{d}w_t + c\,\frac{2\,\Gamma\!\left(\frac{3}{2}\right)}{\sqrt{\pi}} - c. \tag{20}$$

But $2\,\Gamma(3/2)/\sqrt{\pi} = 1$, so

$$\left.\frac{\partial \ln \lambda_s}{\partial \sigma}\right|_{\substack{\mu=0\\ \sigma=1}} = a_2 \left[f_s \int_{-\infty}^{b} (-w_t + b)^{\alpha} \left(w_t^2 - 1\right) \varphi(w_t|\mu = 0, \sigma = 1) \, \mathrm{d}w_t \right.$$

$$\left. + \int_{b}^{\infty} (w_t - b)^{\alpha} \left(w_t^2 - 1\right) \varphi(w_t|\mu = 0, \sigma = 1) \, \mathrm{d}w_t \right]. \qquad (21)$$

Therefore, the sensitivity of $\ln \lambda_s$ to changes in σ depends on a_2, f_s, b, and α. Because the variance of the environment is the square of the standard deviation of the environment, sensitivities of $\ln \lambda_s$ to changes in the variance of the environment can be computed straightforwardly from the results above using the chain rule. We use sensitivities to changes in standard deviation because they have the same units as sensitivities to changes in the mean of the environment, and this is necessary for comparison of the sensitivities. Some thoughts on how results can extend to other parameterizations of w_t and p are in Section S7.

Section S5 Analysis of climate data

The United States Historical Climatology Network database (USHCN [3, 4]) consists of monthly values of precipitation, and minimum, maximum and average temperature from 1221 weather stations from the conterminous United States. The data had been corrected to account for various historical changes in station location, instrumentation, and observing practice; and temperatures (but not precipitation) had been adjusted for time-of-observation bias (due to the 24-hour observation period beginning and ending at times other than local midnight at different stations [5]). Data had been tested for homogeneity by testing for changepoints using a 'pairwise' homogenization algorithm, as described in [6]. Estimates for missing data had been calculated using a weighted average of values from highly correlated neighbouring values [3], providing time series that are complete. All corrections had been performed by climate researchers prior to our use of the database and are described in the data documentation.

The weather data in the USHCN consist of monthly values. These data were preprocessed to derive several variables, all with a sampling frequency of one per year. We chose example aggregations that are likely to be biologically meaningful to populations living in temperate latitudes. *Mean summer temperatures* were obtained by taking the mean of the three mean monthly temperature values corresponding to summer (June-August). *Minimum winter temperature* is the minimum monthly temperature registered during the three winter months (December-February) and *maximum summer temperature* is the maximum monthly temperature during summer. Finally, *total spring precipitation* is the total precipitation during the three spring months (March-May).

Section S6 Special case with $\alpha = 2$ and $a_1 = a_2 = a$

Let $\alpha = 2$ and $a_1 = a_2 = a$. Then,

$$\ln \lambda_s = a \int_{-\infty}^{\infty} (w_t - b)^2 \, \varphi(w_t | \mu = 0, \sigma = 1) \, \mathrm{d}w_t + c \tag{22}$$

$$= a \int_{-\infty}^{\infty} w_t^2 \, \varphi(w_t | \mu = 0, \sigma = 1) \, \mathrm{d}w_t - 2\, a\, b \int_{-\infty}^{\infty} w_t \, \varphi(w_t | \mu = 0, \sigma = 1) \, \mathrm{d}w_t$$

$$+ a\, b^2 + c. \tag{23}$$

By lemma S3.2,

$$\ln \lambda_s = a \, \frac{2\Gamma\left(\frac{3}{2}\right)}{\sqrt{\pi}} + a\, b^2 + c \tag{24}$$

$$= a\,(b^2 + 1) + c. \tag{25}$$

Turning to the sensitivity with respect to μ,

$$\left.\frac{\partial \ln \lambda_s}{\partial \mu}\right|_{\substack{\mu=0\\ \sigma=1}} = a \int_{-\infty}^{\infty} (w_t - b)^2 \, w_t \, \varphi(w_t | \mu = 0, \sigma = 1) \, \mathrm{d}w_t \tag{26}$$

$$= a \int_{-\infty}^{\infty} w_t^3 \, \varphi(w_t | \mu = 0, \sigma = 1) \, \mathrm{d}w_t - 2\, a\, b \int_{-\infty}^{\infty} w_t^2 \, \varphi(w_t | \mu = 0, \sigma = 1) \, \mathrm{d}w_t$$

$$+ a\, b^2 \int_{-\infty}^{\infty} w_t \, \varphi(w_t | \mu = 0, \sigma = 1) \, \mathrm{d}w_t \tag{27}$$

$$= -2\, a\, b \, \frac{2\,\Gamma\left(\frac{3}{2}\right)}{\sqrt{\pi}} = -2\, a\, b. \tag{28}$$

The sensitivity of $\ln \lambda_s$ to changes in σ is

$$\left.\frac{\partial \ln \lambda_s}{\partial \sigma}\right|_{\substack{\mu=0\\ \sigma=1}} = a \int_{-\infty}^{\infty} (w_t - b)^2 \, (w_t^2 - 1) \, \varphi(w_t | \mu = 0, \sigma = 1) \, \mathrm{d}w_t \tag{29}$$

$$= a \int_{-\infty}^{\infty} \left(w_t^4 - 2\, b\, w_t^3 - w_t^2 + b^2\, w_t^2 + 2\, b\, w_t - b^2\right) \varphi(w_t | \mu = 0, \sigma = 1) \, \mathrm{d}w_t. \tag{30}$$

But by lemma S3.2,

$$\left.\frac{\partial \ln \lambda_s}{\partial \sigma}\right|_{\substack{\mu=0\\ \sigma=1}} = a \int_{-\infty}^{\infty} \big(w_t^4 + (b^2-1)\, w_t^2\big)\, \varphi(w_t|\mu=0, \sigma=1)\, \mathrm{d}w_t - a\, b^2 \tag{31}$$

$$= a \int_{-\infty}^{\infty} w_t^4\, \varphi(w_t|\mu=0, \sigma=1)\, \mathrm{d}w_t$$

$$+ a\,(b^2-1) \int_{-\infty}^{\infty} w_t^2\, \varphi(w_t|\mu=0, \sigma=1)\, \mathrm{d}w_t - a\, b^2 \tag{32}$$

$$= a\, \frac{4\,\Gamma\big(\frac{5}{2}\big)}{\sqrt{\pi}} + a\,(b^2-1) - a\, b^2 = 2\, a. \tag{33}$$

The ratio of sensitivities is

$$\frac{\left.\dfrac{\partial \ln \lambda_s}{\partial \mu}\right|_{\substack{\mu=0\\ \sigma=1}}}{\left.\dfrac{\partial \ln \lambda_s}{\partial \sigma}\right|_{\substack{\mu=0\\ \sigma=1}}} = \frac{-2\, a\, b}{2\, a} = -b. \tag{34}$$

Section S7 General p and w_t

How might results be affected by alternative parameterizations of w_t and p? We have already used fairly general parameterizations of both w_t and $p(w_t)$, probably general enough to accommodate most environmental variables and most empirically described response functions in the literature for annual environmental variables and population data and models. But our logic also indicates a straightforward recipe by which other parameterizations could be considered, made explicit here.

If $\varphi(w_t|\mu, \sigma)$ is an arbitrary family of probability density functions (pdfs) parameterized by a mean, μ, and a standard deviation, σ (in the main text and elsewhere in this Supporting Information, normal φ is reasonably assumed, since most weather variables will be reasonably transformable to normal), and if $p(w_t)$ is an arbitrary log response function, then

$$\left.\frac{\partial \ln \lambda_s}{\partial \mu}\right|_{\substack{\mu=0\\ \sigma=1}} = \int_{-\infty}^{\infty} p(w_t)\, \frac{\partial}{\partial \mu}\, \varphi(w_t|\mu, \sigma)\Big|_{\substack{\mu=0\\ \sigma=1}} dw_t \tag{35}$$

and

$$\left.\frac{\partial \ln \lambda_s}{\partial \sigma}\right|_{\substack{\mu=0\\ \sigma=1}} = \int_{-\infty}^{\infty} p(w_t)\, \frac{\partial}{\partial \sigma}\, \varphi(w_t|\mu, \sigma)\Big|_{\substack{\mu=0\\ \sigma=1}} dw_t. \tag{36}$$

It is difficult to judge the relative magnitudes of these quantities without specifying parameterizations for φ and p, but it would be easy to do so following the same procedures used in this study for any proposed parameterizations. For the case of

normal φ, the above equations reduce to

$$\left.\frac{\partial \ln \lambda_s}{\partial \mu}\right|_{\substack{\mu=0\\ \sigma=1}} = \int_{-\infty}^{\infty} p(w_t)\, w_t\, \varphi(w_t|\mu=0,\sigma=1)dw_t \tag{37}$$

$$= \int_{0}^{\infty} (p(w_t)-p(-w_t))\, w_t\, \varphi(w_t|\mu=0,\sigma=1)dw_t \tag{38}$$

and

$$\left.\frac{\partial \ln \lambda_s}{\partial \sigma}\right|_{\substack{\mu=0\\ \sigma=1}} = \int_{-\infty}^{\infty} p(w_t)\, (w_t^2-1)\, \varphi(w_t|\mu=0,\sigma=1)dw_t \tag{39}$$

$$= \int_{0}^{\infty} (p(w_t)+p(-w_t))(w_t^2-1)\, \varphi(w_t|\mu=0,\sigma=1)dw_t. \tag{40}$$

For any given parameterization of $p(w_t)$ it is easy to numerically compute how these integrals depend on the parameters, and then to see how the signs of the sensitivities and the relative magnitudes of the sensitivities depend on parameters.

Response functions of some environmental variables may seem likely to be "saturating" rather than peaked, i.e., vital rates continually increase with more and more extreme values of the environmental variable, approaching an asymptote. Such scenarios could be straightforwardly analyzed with the above equations. However, our view is that such scenarios can usually or always be more appropriately modelled with a peaked response function, with the peak far from the values manifested by the local environment. Food level, if considered an environmental variable, would have a saturating response function. Only physical environmental variables were considered in this study. Food level would often be affected by the focal population as well as affecting it, and so should probably be modelled as a state variable interacting with the population level, rather than as an external, "environmental" variable. Food levels may be considered an external variable if they are not affected by the focal population, but instead are controlled entirely by rainfall, for instance. But then the environmental variable used should perhaps be rainfall, not food. There will be a peak in the response function written as a function of rainfall, because excessive rainfall as well as insufficient rainfall will cause reduced food levels, which will impact population vital rates. In some areas, rainfall will never reach or exceed levels that cause this peak to be attained or passed, but the peak still exists in principle even if it does not manifest in a given locale.

Response functions with thresholds may also seem worth considering, and could also be analyzed straightforwardly with the above equations. But again, in our view, threshold response functions are of secondary importance for an initial theoretical study such as this one. They seem unlikely for annually censused populations, the most common sampling setup for demographic models and data. Whether the current temperature is below or above freezing, for instance, may certainly affect organism performance in the short-term. But annualized temperature averages over a period (e.g., winter average temperature, or spring average temperature), or other seasonal summary quantities (degree days, or number of days with frost), seem more likely to be the environmental inputs into demographic models such as the model we consider, and seem more likely to be statistically related to annually measured vital rates. Variables of this kind also seem less likely to exhibit clear and discontinuous thresholds.

Dell *et al.* (2011; [7]) analyzed 1072 thermal responses for 309 species of microbes, plants, and animals, attempting to understand what response functions look like over the full range of values of the temperature variables. Out of the 1072 thermal responses examined, 26 were specifically on population growth rates, including 14 terrestrial, nine freshwater, and three marine populations. Of these 26 response functions, 18 included an optimum temperature, and the remaining ones may not have included sufficient data extent to show the optimum if it existed. For this reason, the authors "encourage experimentalists to measure the response ... over the full temperature range, thus allowing characterization of the entire unimodal response" [7]. Thomas *et al.* (2012; [8]) examined growth responses to temperature in more than 130 species of phytoplankton. The authors state that "Two features of [response functions] are common to all ectotherms: unimodality and negative skewness". Huey & Berrigan ([9]; 2001) collected data on 78 species, which mostly included Arthropoda, but also a fungus and two worms. Analysing the shape of response functions of population growth rate was not one of their objectives. However, out of the six example response functions they provide, five include an optimum temperature, the exception being a polychaete that shows a monotonic function within the temperature range investigated (14°C to 28°C), but that may show a peak outside that range. In another metastudy, Deutsch *et al.* (2008; [10]) looked at response functions of population growth rate to temperature for 38 species of insects. They fitted observed growth rates at multiple temperatures to a Gaussian function to describe the rise in performance up to an optimum temperature, and a quadratic decline to zero at higher temperatures (a left-skewed unimodal function, similar to one that can be produced with our approach using $f_s < 0$). The presence of an optimum temperature was taken for granted. Jenouvrier *et al.* ([11]; 2012) investigated the potential effects of predicted climate change on an emperor penguin population in Antarctica. For emperor penguins, an intermediate sea-ice concentration (SIC: the fraction of the study area covered by sea ice) represents the ideal environment. Both an absence of sea-ice (due to a lack of habitat), and heavy, persistent sea ice (because of longer foraging trips, higher energetic costs, and lower provisioning of chicks) reduce population fitness (as measured by the population's long-term stochastic growth rate; $\ln \lambda_s$). Theoretical support was also provided by Amarasekare & Savage (2012; [12]), who showed that a peaked response function was to be expected.

Section S8 Additional climate data analysis results

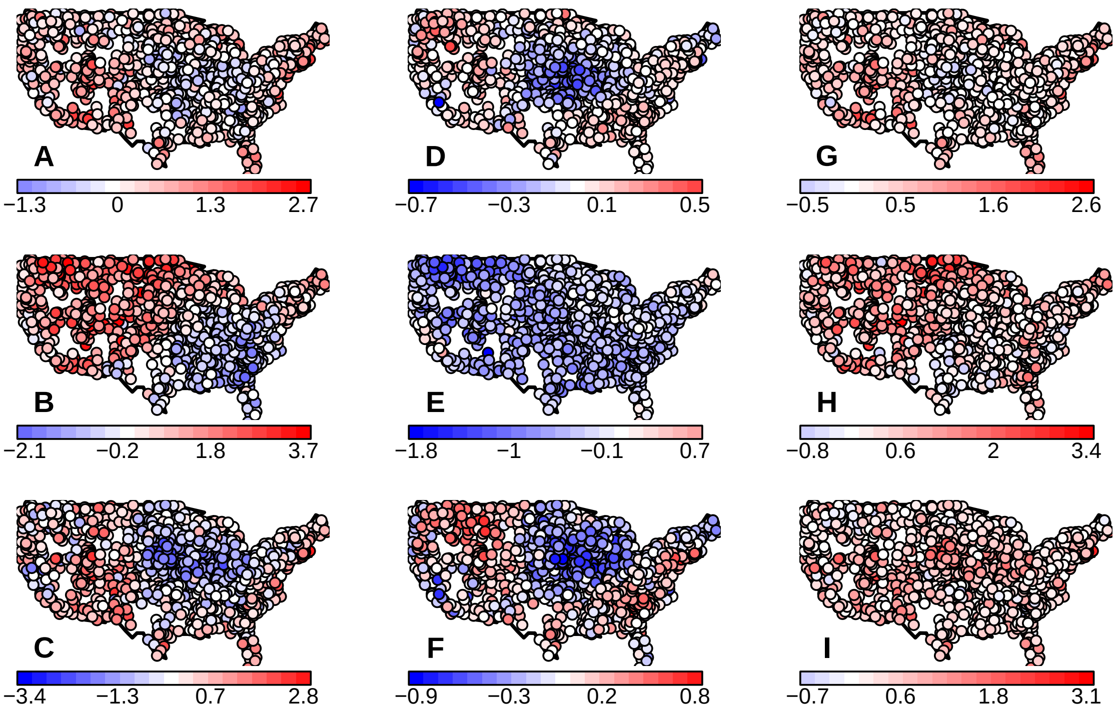

Figure S1: Relative changes in mean and standard deviation of climate variables in the United States. If m_1 and sd_1 are the mean and standard deviation of summer temperature in a location for the period 1911-1945, and m_2 and sd_2 are the mean and standard deviation of summer temperature in the same location for the period 1976-2010, then: panel A shows $m_2 - m_1$, the degree of change in environmental mean; panel D shows $sd_2 - sd_1$, the degree of change in environmental standard deviation; and panel G shows $|m_2 - m_1| - |sd_2 - sd_1|$, which indicates the relative magnitudes of these changes and is positive when changes in mean exceed changes in standard deviation and negative otherwise. White corresponds to no change on A-D and to equal changes in mean and standard deviation on E-F. Environmental variables depicted are summer mean temperature (A, D, G), winter minimum temperature (B, E, H), and summer maximum temperature (C, F, I).

Section S9 Semelparous populations

Our results apply not only to unstructured populations, but also to semelparous populations. Consider a semelparous population with k age classes with a transition matrix

$$\mathbf{\Lambda}_t = \begin{bmatrix} 0 & \cdots & g(t) \\ s_1(t) & \cdots & 0 \\ \cdots & s_{k-1}(t) & 0 \end{bmatrix},$$

where the $s(t)$ are the survival rates for each age class and $g(t)$ is the fertility rate for the final, reproductive age class. The product of k matrices like the one above yields a diagonal matrix model equivalent to our model [13].

References

[1] Abramowitz M, Stegun IA, editors (1964) Handbook of Mathematical Functions with Formulas, Graphs, and Mathematical Tables. Dover publications.

[2] Gradshteyn IS, Ryzhik IM (2007) Table of Integrals, Series, and Products. Oxford: Academic Press, seventh edition.

[3] Menne MJ, Williams Jr CN, Vose RS (2009) The U.S. Historical Climatology Network monthly temperature data, version 2. *Bull Amer Meteor Soc* 90(7): 993–1007. doi:10.1175/2008BAMS2613.1.

[4] National Climatic Data Center, National Oceanic and Atmospheric Administration (2011). The USHCN Version 2 Serial Monthly Dataset. `ftp://ftp.ncdc.noaa.gov/pub/data/ushcn/v2/monthly/`. Accessed on 16/04/2011.

[5] Vose RS, Williams Jr CN, Peterson TC, Karl TR, Easterling DR (2003) An evaluation of the time of observation bias adjustment in the U. S. Historical Climatology Network. *Geophys Res Lett* 30(20): 2046. doi:10.1029/2003GL018111.

[6] Menne MJ, Williams Jr CN (2009) Homogenization of temperature series via pairwise comparisons. *J Climate* 22(7): 1700–1717. doi:10.1175/2008JCLI2263.1.

[7] Dell AI, Pawar S, Savage VM (2011) Systematic variation in the temperature dependence of physiological and ecological traits. *Proc Natl Acad Sci U S A* 108(26): 10591–10596. doi:10.1073/pnas.1015178108.

[8] Thomas MK, Kremer CT, Klausmeier CA, Litchman E (2012) A global pattern of thermal adaptation in marine phytoplankton. *Science* 338(6110): 1085–1088. doi:10.1126/science.1224836.

[9] Huey RB, Berrigan D (2001) Temperature, demography, and ectotherm fitness. *Am Nat* 158(2): 204–210. doi:10.1086/321314.

[10] Deutsch CA, Tewksbury JJ, Huey RB, Sheldon KS, Ghalambor CK, *et al* (2008) Impacts of climate warming on terrestrial ectotherms across latitude. *Proc Natl Acad Sci U S A* 105(18): 6668–6672. doi:10.1073/pnas.0709472105.

[11] Jenouvrier S, Holland M, Stroeve J, Barbraud C, Weimerskirch H, *et al* (2012) Effects of climate change on an emperor penguin population: analysis of coupled demographic and climate models. *Glob Change Biol* 18(9): 2756–2770. doi:10.1111/j.1365-2486.2012.02744.x.

[12] Amarasekare P, Savage V (2012) A framework for elucidating the temperature dependence of fitness. *Am Nat* 179(2): 178–191. doi:10.1086/663677.

[13] Tuljapurkar SD (1990) Population Dynamics in Variable Environments. New York, NY: Springer-Verlag.
